# Association Between the Retinal Biomarkers and Cognitive Impairment in Community‐dwelling Older Adults in Taiwan

**DOI:** 10.1002/alz.087811

**Published:** 2025-01-09

**Authors:** Ting‐Wen Chu, Yi‐Ting Hsieh, Jeng‐Min Chiou, Yao‐Lin Liu, Jen‐Hau Chen, Yen‐Ching Chen

**Affiliations:** ^1^ Institute of Epidemiology and Preventive Medicine, College of Public Health, National Taiwan University, Taipei Taiwan; ^2^ Department of Ophthalmology, Mackay Memorial Hospital, Taipei Taiwan; ^3^ Department of Medicine, MacKay Medical College, New Taipei City Taiwan; ^4^ Department of Ophthalmology, National Taiwan University Hospital, Taipei Taiwan; ^5^ Institute of Statistics and Data Science, National Taiwan University, Taipei, Taipei Taiwan; ^6^ Department of Ophthalmology, Far Eastern Memorial Hospital, New Taipei City Taiwan; ^7^ Department of Geriatrics and Gerontology, National Taiwan University Hospital, No. 1, Changde Street, Taipei Taiwan; ^8^ College of Medicine, National Taiwan University, Taipei Taiwan; ^9^ College of Public Health, National Taiwan University, Taipei City, Taipei Taiwan

## Abstract

**Background:**

The early detection of preclinical dementia is crucial, prompting investigations into retinal biomarkers using optical coherence tomography (OCT). Inconsistent and limited longitudinal studies have been done to clarify the association between the retinal nerve fiber layer (RNFL) and ganglion cell‐inner plexiform layer (GC‐IPL) thickness and cognitive function over time. This study aims to explore the association between retinal biomarkers and cognitive function over time in non‐demented older adults.

**Method:**

This seven‐year prospective cohort study included 264 non‐demented older adults at baseline (2015‐2017) from the ongoing Taiwan Initiative for Geriatric Epidemiological Research. Cognitive function underwent biennial follow‐ups to 2022, with assessments including global and domain‐specific cognition (memory, attention, executive function, and verbal fluency) measured using the Montreal Cognitive Assessment–Taiwanese version (MoCA‐T) and a battery of neuropsychological tests. Retinal data were collected using OCT at baseline. Generalized linear mixed models were utilized to examine the relationship between the RNFL, GC‐IPL thickness, and cognitive function, adjusting for apolipoprotein E ε4 status, age, sex, education years, and age‐related macular degeneration.

**Result:**

At baseline, the performance of global cognition (MoCA‐T) decreased as GC‐IPL deviated from the mean (77.1 μm) (quadratic GC‐IPL: β= ‐0.32 x 10^‐2^; 95% CI: ‐0.61 x 10^‐2^ to ‐0.03 x 10^‐2^). Similar findings were found for memory performance (quadratic GC‐IPL: β= ‐0.19 x 10^‐2^; 95% CI: ‐0.31 x 10^‐2^ to ‐0.06 x 10^‐2^), and executive function (quadratic GC‐IPL: β= ‐0.07 x 10^‐2^; 95% CI: ‐0.14 x 10^‐2^ to ‐0.003 x 10^‐2^) over seven years. These results remained significant in females after stratification by sex. Notably, no significant association was observed between RNFL thickness and cognition.

**Conclusion:**

We found a non‐linear relation between GC‐IPL thickness and poor performance of global cognition, memory, and executive function, particularly among women. These findings highlight the significance of GC‐IPL thickness as an early biomarker of cognitive impairment, informing strategies for timely intervention in aging populations.

